# Development of a uniform, very aggressive disease phenotype in all homozygous carriers of the *NOD2* mutation p.Leu1007fsX1008 with Crohn’s disease and active smoking status resulting in ileal stenosis requiring surgery

**DOI:** 10.1371/journal.pone.0236421

**Published:** 2020-07-27

**Authors:** Fabian Schnitzler, Matthias Friedrich, Marianne Angelberger, Julia Diegelmann, Johannes Stallhofer, Christiane Wolf, Joel Dütschler, Samuel Truniger, Torsten Olszak, Florian Beigel, Cornelia Tillack, Peter Lohse, Stephan Brand

**Affiliations:** 1 Department of Medicine II—Grosshadern, Ludwig-Maximilians-University (LMU) Munich, Munich, Germany; 2 Nuffield Department of Orthopaedics, Kennedy Institute of Rheumatology, Rheumatology and Musculoskeletal Sciences, University of Oxford, Oxford, United Kingdom; 3 Department of Preventive Dentistry and Periodontology, LMU Munich, Munich, Germany; 4 Department of Medicine IV, University Hospital, Friedrich-Schiller University, Jena, Germany; 5 Max-Planck-Institute of Psychiatry, Munich, Germany; 6 Department of Gastroenterology and Hepatology, Kantonsspital St. Gallen, St. Gallen, Switzerland; 7 Institute of Laboratory Medicine and Human Genetics, Singen, Germany; Unicamillus, Saint Camillus International University of Health Sciences, ITALY

## Abstract

**Background:**

*NOD2* variants are the strongest genetic predictors for susceptibility to Crohn’s disease (CD). However, the clinical value of *NOD2* on an individual patient level remains controversial. We aimed to define the predictive power of the major *NOD2* mutations regarding complicated CD in a large single center cohort.

**Methods:**

1076 CD patients were prospectively genotyped for the three common CD-associated *NOD2* mutations rs2066844, rs2066845, and rs2066847, followed by detailed genotype-phenotype analyses.

**Results:**

Overall, 434 CD patients (40.3%) carried at least one of the three main *NOD2* mutations. A significantly higher minor allele frequency (15.6%) of the *NOD2* frameshift mutation p.Leu1007fsX1008 (rs2066847) was seen in patients with aggressive disease compared to 8.2% in patients with mild disease (p = 2.6 x 10^−5^). Moreover, a total of 54 CD patients (5.0%) were homozygous for this *NOD2* frameshift mutation. 100% of these patients had ileal disease compared to 82% of *NOD2* wild-type carriers (p<0.0001). In homozygous carriers of the *NOD2* frameshift mutation, 87% presented with ileal stenosis, 68.5% had fistulas, and 72.2% required CD-related surgery despite immunosuppressive therapy in 87% of these patients. All homozygous carriers of the 1007fs mutation who were active smokers had ileal stenosis and required CD-related surgery.

**Conclusion:**

Homozygosity for Leu1007fsX1008 is an excellent biomarker for predicting complicated CD on an individual patient level. Active smoking and homozygosity for this mutation is associated with a 100% risk for developing ileal stenosis requiring CD-related surgery. In these patients, smoking cessation and early initiation of immunosuppressive strategies may be beneficial.

## Introduction

The identification of the first genetic locus in the centromeric region of chromosome 16 associated with susceptibility to inflammatory bowel diseases (IBD) more than two decades ago provided first insights into the role of a genetic predisposition in the pathogenesis of IBD [1]. In this chromosomal region, *NOD2* was identified five years later as the first gene to be associated with susceptibility to Crohn’s disease (CD) [2,3]. Three main *NOD2* mutations, p.Arg702Trp (rs2066844), p.Gly908Arg (rs2066845), and p.Leu1007fsX1008 (rs2066847), are associated with a complicated disease course, particularly with a stricturing and penetrating disease behaviour [2–4]. Since 2001, several genetic studies including genome-wide association scans (GWAS) unrevealed the complex genetic background of the two IBD subtypes CD and ulcerative colitis (UC) [5–22]. Currently, more than 250 susceptibility regions for IBD have been confirmed, but only *NOD2* has evolved from an initial candidate gene to a clinically useful genetic marker for disease prediction in CD [22,23].

In particular, a frameshift mutation in exon 11 of *NOD2*, p.Leu1007fsX1008 (rs2066847), has been successfully used as a predictive factor for therapeutic decisions in patients with CD [7,8]. Several clinical trials reported on CD patients with homozygosity for p.Leu1007fsX1008. Due to the limited number of patients in these subcohorts, the statistical power to reliably predict the disease course of CD was too low [12–15]. In 2006, we reported a subgroup of 19 patients with homozygosity for p.Leu1007fsX1008 in our IBD cohort [8]. At that time, this study represented the largest subgroup analysis of patients with p.Leu1007fsX1008 homozygosity and the respective phenotypes. Importantly, this patient population was characterized by an early onset of CD with a median age at diagnosis of 23.9 years and 100% ileal disease. Ninety-five percent of the patients had stricturing disease behaviour with long-segment stenosis in the terminal ileum, entero-enteral fistulas (52.6%), and the frequent need for CD-related surgery (73.7%) with a high prevalence of re-stenosis at the anastomosis (78.6%).

However, the value of *NOD2* on an individual patient level remains controversial. In this study, we aimed to increase the CD patient cohort of homozygous carriers of the *NOD2* mutation p.Leu1007fsX1008 in order to evaluate its predictive power for the CD disease course. We genotyped 1076 CD patients for the three main *NOD2* mutations p.Arg702Trp (rs2066844), p.Gly908Arg (rs2066845), and p.Leu1007fsX1008 (rs2066847) and identified a total of 54 homozygous rs2066847 minor C allele carriers. The predictive power of the three common *NOD2* mutations was subsequently analyzed by detailed genotype-phenotype correlations.

## Patients and methods

### Ethics statement

This study was approved by the ethics committee of the medical faculty of the Ludwig-Maximilians-University Munich. Written, informed consent was obtained from all patients prior to study inclusion. Study protocols were based on the ethical principles for medical research involving human subjects of the Helsinki Declaration.

### Study population

In total, 1076 patients with CD were enrolled in this study. All patients were recruited at the University Hospital Munich-Grosshadern. Our study cohort included a predominantly Caucasian population (99.5% Caucasians, only 0.5% (n = 5 patients) were from India or of African descent).

Phenotypic parameters were collected independently of the results of the genotype analyses. Phenotypic data included demographic and clinical data (behaviour and anatomic location of IBD, disease-related complications, surgical or immunosuppressive therapy). Two senior gastroenterologists analyzed the data which were recorded by patient chart analysis and a detailed questionnaire based on an interview at the time of enrolment. For the analysis of demographic and phenotypic data, the diagnosis of CD was related to established international guidelines based on endoscopic, radiological, and histopathological parameters [24]. CD patients were classified according to the Montreal classification [25] including age at diagnosis (A), location (L), and behaviour (B) of disease.

The clinical course of CD was defined as “aggressive” in CD patients with a stricturing (B2) and/or penetrating disease behaviour (B3) and/or CD-related surgery. CD patients with a “mild” disease course did not have a stricturing or penetrating phenotype or CD-related surgery (B1 according to the Montreal Classification) [25].

### DNA extraction and *NOD2* genotyping

Amplification of *NOD2* exons 4, 8, and 11 was performed according to standard procedures. PCR product size and quantity were analyzed by agarose gel electrophoresis. Fragments were purified with the QIAquick PCR purification kit (QIAGEN, Hilden, Germany) and sequenced with the ABI PRISM BigDye Terminator v3.1 Ready Reaction Cycle Sequencing kit (Applied Biosystems, Foster City, CA, USA). Sequences were analyzed on an ABI PRISM 377 DNA Sequencer (Applied Biosystems) using the Sequence Analysis program version 3.4.5 (Applied Biosystems). Control subject and patient sequence data were compared to the published *NOD2* sequence, and all differences were documented. Different analyses of this IBD patient cohort have been previously published elsewhere [7–9,26–28].

### Statistical analyses

All statistical tests were two-tailed and p-values < 0.05 were considered significant. Each genetic marker was tested for Hardy-Weinberg equilibrium. Single-marker allelic tests were performed with Pearson’s χ2 test. Odds ratios were calculated for the minor allele at each SNP. For evaluation of phenotypic consequences, we conducted logistic regression analyses. Data and haplotype analyses were done by using the PLINK v1.07 software (http://pngu.mgh.harvard.edu/purcell/plink/) [29] and R-2.4.1. (http://cran.r-project.org).

The combined effects of five important predictors (age at diagnosis, disease duration, smoking status, ileal involvement and homozygosity for p.Leu1007fsX1008 *NOD2* mutation (rs2066847)) on the presence of stenoses and on the need for surgery were analyzed with multiple logistic regression. The models were fitted through a Bayesian approach with a weakly informative prior using the function “bayesglm” in the R package arm [30]. This approach was needed because of fitting problems due to quasi-complete separation for the effect of the p.Leu1007fsX1008 *NOD2* mutation (rs2066847) on stenoses. For all other predictors in the model and for the effect of the p.Leu1007fsX1008 *NOD2* mutation on surgery, p-values and odds ratios were closely similar with this approach as with conventional logistic regression (function “glm” in R base package stats). Odds ratios were calculated from the regression coefficients, and approximate 95% CI were calculated from 95% Wald confidence intervals as obtained with the function “coefci” in R package lmtest. To obtain more interpretable odds ratios, coefficients for age and disease duration were calculated for an increase by 10 years. Smoking (with three levels) was represented either by a single contrast (any smoking vs. non-smoking) or by two separate contrasts (active smoking vs. non-smoking and former smoking vs. non-smoking).

## Results

### Phenotypic characteristics of the study cohort

S1 Table shows the phenotypic characteristics of the study population. A total of 1076 CD patients were included in the analysis. There was an equal gender distribution (48.0% males, 52.0% females), and the mean age was 42.3 years with a mean disease duration of almost 12 years. The majority of CD patients (71.6%) was between 17 and 40 years of age at first diagnosis of CD, while 16.1% of the CD patients were younger than 17 years at the time of CD onset. 72.6% of the patients studied had either a stricturing or a penetrating disease behaviour (26.8% stricturing disease behaviour (B2); 45.8% penetrating disease behaviour (B3)), thus representing a CD cohort with a severe disease manifestation. 57.0% of the patients required CD-related surgery (e.g., ileocecal resection, fistulectomy, colectomy, or ileostomy). Over 80% of the CD patients were treated with immunosuppressive or biological therapies (S1 Table). Interestingly, almost one fifth of the patients (17.7%) had a positive family history of IBD, illustrating a strong genetic predisposition in this patient population. Importantly, more than one third of the CD patients (35.5%) were active smokers and more than one fifth were ex-smokers (21.8%) at the time of enrolment.

### Allele frequencies of the three main *NOD2* mutations rs2066844 (p.Arg702Trp), rs2066845 (p.Gly908Arg) and rs2066847 (p.Leu1007fsX1008)

Table 1 gives an overview of the observed minor allele frequencies and the genotype of the three main *NOD2* mutations rs2066844 (p.Arg702Trp), rs2066845 (p.Gly908Arg), and rs2066847 (p.Leu1007fsX1008) in our CD patient cohort.

**Table 1:** Minor allele frequencies (MAF) of the three main *NOD2* SNPs rs2066844 (p.Arg702Trp), rs2066845 (p.Gly908Arg), and rs2066847 (p.Leu1007fsX1008) for each genotype (homozygous, heterozygous and compound heterozygous) in patients with Crohn’s disease.

Crohn’s disease		
Gene marker	Genotype	MAF
		(%)
rs2066844 (n = 1076)		
p.Arg702Trp, minor allele	T	8.0 (n = 172/2152)
Homozygous	TT	1.1 (n = 24/2152)
Heterozygous	CT	5.3 (n = 114/2152)
Compound heterozygous	CT/other *NOD2* mutation	1.6 (n = 34/2152)
rs2066845 (n = 1076)		
p.Gly908Arg, minor allele	C	3.9 (n = 84/2152)
Homozygous	CC	0.2 (n = 4/2152)
Heterozygous	GC	0.5 (n = 68/2152)
Compound heterozygous	GC/other *NOD2* mutation	3.2 (n = 12/2152)
rs2066847 (n = 1066)		
p.Leu1007fsX1008, minor allele	insC	13.4 (n = 286/2132)*
Homozygous	CC	5.0 (n = 108/2132)*
Heterozygous	XC	7.2 (n = 153/2132)*
Compound heterozygous	XC/other *NOD2* mutation	1.2 (n = 25/2132)*

*For rs2066847 (p.Leu1007fsX1008), genotype status was only known in 1066 patients, resulting in 2132 alleles.

The observed minor allele frequencies of 8.0% (p.Arg702Trp), 3.9% (p.Gly908Arg), and 13.4% (p.Leu1007fsX1008) were comparable to previously reported allele frequencies, e.g., in a French IBD population [17]. 59.7% of the CD patients analyzed (n = 642) carried none of the three main *NOD2* mutations, whereas 40.3% (n = 306) were carriers of one (or more) *NOD2* variants. Of the 306 patients carrying *NOD2* mutations, 28.4% were heterozygous carriers of one of the three main *NOD2* mutants, 4.6% were compound heterozygous carriers, and 6.3% (n = 68) were homozygous (n = 12 with homozygosity for p.Arg702Trp, n = 2 with homozygosity for p.Gly908Arg, and n = 54 with homozygosity for p.Leu1007fsX1008, Table 1). Genotype frequencies for the *NOD2* mutations were as follows: 59.7% were wildtype for *NOD2* mutations, 16.1% were genotyped for rs2066844, 7.8% for rs2066845 and 26.8% for rs2066847 (Please note: Due to compound heterozygous carriers (see for details Table 1), the total percentage is > 100%; the genotype status of rs20066847 was known in n = 1066 CD patients).

### *NOD2* genotype-phenotype correlation in patients with Crohn’s disease demonstrates a gene-dosage effect of *NOD2* mutations and reveals p.Leu1007fsX1008 as a highly predictive genetic marker for severe Crohn’s disease

Detailed genotype-phenotype analyses demonstrated a strong association between the number of affected alleles and the severity of Crohn’s disease in our CD cohort. Carriers with two *NOD2* mutations, e. g., compound heterozygous and homozygous carriers, were significantly younger at the first diagnosis of IBD (p = 0.010 and p = 0.036, respectively) than *NOD2* wild-type carriers (“wild-type” defined as the absence of the three main *NOD2* mutations. There was no significant difference seen in patients with one affected allele with respect to age at diagnosis.

More patients with one or two altered *NOD2* alleles had ileal involvement compared to *NOD2* wild-type (95.9% of compound heterozygotes, 94.2% of homozygotes and 89.8% of *NOD2* heterozygous mutation carriers compared to 82.0% of patients with the *NOD2* wild-type, p = 0.318, p = 0.048 and p = 0.002 compared to 89.2% of patients with the *NOD2* wild-type and ileal involvement).

Significant more compound heterozygous and homozygous *NOD2* mutation carriers had a penetrating disease behavior (B3) compared to *NOD2* wild-type patients (59.2% and 64.7% vs. 44.3%, respectively, p = 0.047 and p = 0.0003 compared to *NOD2* wild-type carriers).

Contrarily, more *NOD2* wild-type (29.6%) and heterozygous carriers (29.0%) had a non-stricturing and non-penetrating disease behavior (B1) compared to 14.3% and 10.3% of patients with compound heterozygosity and homozygosity for *NOD2* mutations (p = 0.014 and p = 0.016, respectively; and p = 0.001 compared to 10.3% with B1 in homozygous carriers). In addition, for stenoses, fistulas, and need for CD-related surgery, there was a gene dosage with a more severe disease phenotype found amongst the 1007fs homozygous carriers.

No significant differences were seen for gender distribution, disease duration, body mass index (BMI), perianal fistulas, and the number of patients with a positive smoking history between the groups. No significant differences were also observed for p.Arg702Trp (rs2066844) and for p.Gly908Arg (rs2066845) regarding allele frequencies in CD patients with aggressive disease compared to mild CD (Table 2). In contrast, a higher minor allele frequency (15.6%) of the p.Leu1007fsX1008 (rs2066847) frameshift mutation was seen in patients with aggressive disease, compared to 8.2% in patients with mild disease which was highly significant (p = 2.63x10^-5^, Table 2).

**Table 2:** CD phenotype stratified by disease course (n = 1011).

*NOD2*		Aggressive disease behaviour	Mild disease behaviour			
SNP		(stenosis and/or fistula and/or CD-related surgery; n = 747)	(no stenosis, no fistula, no CD-related surgery = inflammatory disease type; n = 264)	p-value	OR	95% CI
**rs2066844**	**T-allele (%)**	**119/1494 (8.0%)**	**49/528 (9.3%)**	0.347	0.850	[0.60–1.20]
	C-allele (%)	1375/1494 (92.0**%**)	479/528 (90.7)			
**rs2066845**	**C-allele (%)**	**64/1494 (4.3%)**	**15/528 (2.8%)**	0.141	1.531	[0.86–2.71]
	G-allele (%)	1430/1494 (95.7**%**)	513/528 (97.2**%**)			
**rs2066847**	**C-allele (%)**	231/1482 (15.6%)*	43/522 (8.2%)*	**2.633x10**^**-5**^	2.057	[1.46–2.90]
	X-allele (%)	1251/1482 (84.4**%**)*	479/522 (91.8**%**)*			

Aggressive disease behaviour was defined as a stricturing and/or penetrating disease course and/or CD-related surgery. Given are the allele frequencies (upper line: minor allele frequency in bold, bottom line: major allele frequency) for CD patients with an aggressive and a mild disease behaviour, respectively, for the three main *NOD2* mutants rs2068844, rs2066845, and rs2066847. Only the frameshift mutation p.Leu1007fsX1008 in exon 11 of the *NOD2* gene (rs2066847) was significantly associated with aggressive disease behaviour in patients with CD (p<0.0001, Chi-squared test). For the other two *NOD2* mutants, no significant differences were seen for the allele frequencies in patients with aggressive vs. mild disease behaviour. Parts of these data have been published elsewhere [8,26,28,31].

Overall, the number of affected alleles was significantly associated with the severity of CD. Compound heterozygous and homozygous carriers of *NOD2* mutations had a more aggressive disease course compared to the *NOD2* wild-type and heterozygous *NOD2* mutation carriers (92.6% of the homozygous carriers, 87.8% of the compound heterozygous carriers vs. 72.0% of patients with *NOD2* wild-type and 79.2% of the heterozygous carriers, p = 0.003, p = 0.023 and p = 0.065 compared to 72%, Table 3).

**Table 3:** CD phenotype stratified by the rs2066847 (p.Leu1007fsX1008) genotype in CD patients.

*NOD2* SNP rs2066847	(1)	(2)	(3)	(1) vs. (3)	(2) vs. (3)
genotype status	Homozygous	Heterozygous	Wild-type	p-value	p-value
	(n = 54)	(n = 178)	(n = 642)	OR (CI)	OR (CI)
**Location**					
	(n = 54)	(n = 171)	(n = 610)		
Terminal ileum (L1)	18 (33.3%)	50 (29.2%)	120 (19.7%)	**0.020**	**0.008**
				2.04	1.69
				[1.12–3.72]	[1.15–2.48]
Colon (L2)	0	10 (5.9%)	98 (16.0%)	0.0002*	0.001*
				0	0.32
				[0–0.38]	[0.17–0.64]
Ileocolon (L3)	36 (66.7%)	110 (64.3%)	380 (62.3%)	0.525	0.626
				1.21	1.09
				[0.67–2.18]	[0.77–1.55]
Upper GI (L4)	7 (13.0%)	26 (15.2%)	90 (14.8%)	0.721	0.884
				0.86	1.04
				[0.38–1.96]	[0.65–1.66]
Any ileal	54 (100%)	160 (93.6%)	500 (82.0%)	<0.0001*	0.0004*
involvement				NA	3.2
(L1+L3)				[3.05—infinite]	[1.68–6.10]
**Behaviour** ^1^					
	(n = 54)	(n = 168)	(n = 589)		
Non-stricturing, Non-penetrat. (B1)	4 (7.4%)	37 (22.0%)	177 (29.6%)	0.001*	**0.043**
				0.19	0.66
				[0.07–0.52]	[0.44–0.99]
Stricturing (B2)	13 (24.1%)	54 (32.2%)	156 (26.1%)	0.700	0.149
				0.88	1.31
				[0.46–1.69]	[0.91–1.91]
Penetrating (B3)	37 (68.5%)	77 (45.8%)	265 (44.3%)	0.001*	0.847
				2.66	1.03
				[1.47–4.83]	[0.73–1.46]
**Surgery because of CD** ^3^					
	(n = 54)	(n = 167)	(n = 575)		
	39 (72.2%)	109 (65.3%)	306 (53.2%)	**0.009**	**0.006**
				2.29	1.65
				[1.23–4.24]	[1.15–2.36]
**Fistulas**					
	(n = 54)	(n = 167)	(n = 590)		
	37 (68.5%)	77 (46.1%)	261 (44.2%)	0.0009*	0.668
				2.74	1.08
				[1.51–4.98]	[0.76–1.52]
**Stenosis**					
	(n = 54)	(n = 164)	(n = 585)		
	47 (87.0%)	109 (66.5%)	336 (57.4%)	0.0001*	**0.038**
				4.98	1.47
				[2.21–11.19]	[1.02–2.11]
**Smoking**					
Active smoker, ex-smoker	(n = 42)	(n = 120)	(n = 372)		
	17 (40.5%)	64 (53.3%)	223 (60.0%)	**0.017**	0.202
				0.45	0.76
				[0.24–0.87]	[0.50–1.16]
**Disease course**					
	(n = 54)	(n = 168)	(n = 597)		
**Aggressive**	50 (92.6%)	133 (79.2%)	430 (72.0%)	**0.003**	0.065
				4.85	1.48
				[1.73–13.65]	[0.98–2.23]
**Mild**	4 (7.4%)	35 (20.8%)	167 (28.0%)	**0.003**	0.065
				0.21	0.68
				[0.07–0.58]	[0.45–1.02]

Group (1): Subcohort of 54 patients with homozygosity for p.Leu1007fsX1008. CD disease characteristics are based on the Montreal classification [25]. For each variable, the number of patients included is given. ^1^Disease behaviour was defined according to the Montreal classification. A stricturing disease phenotype was defined as the presence of stenoses without penetrating disease. The diagnosis of stenoses was made surgically, endoscopically, or radiologically (using MR enteroclysis). ^2^Immunosuppressive agents included azathioprine, 6-mercaptopurine, methotrexate, infliximab and/or adalimumab. ^3^ Only surgery related to CD-specific problems (e.g. fistulectomy, colectomy, ileostomy) was included. Group (2): CD patients heterozygous for the p.Leu1007fsX1008 (rs2066847) mutant (n = 178) including a total of 38 compound heterozygotes (n = 16 for p.Leu1007fsX1008/p.Gly908Arg (rs2066844) and n = 22 for p.Leu1007fsX1008/p.Arg702Trp (rs2066845)). Group (3): CD patients carrying the *NOD2* wild-type (642 patients with none of the three main *NOD2* mutations); group

* Given the large number of comparisons, we performed correction for multiple testing (for a total of n = 28 comparisons). Therefore, the threshold for a significant p-value was < 0.05 x 28 = 0.001786 and only p-values with an asterisk (*) remained significant after multiple testing.

### Sub-analysis of *NOD2* p.Leu1007fsX1008 homozygosity in patients with Crohn’s disease reveals associations with an aggressive disease phenotype characterized by ileal stenosis, fistulas, and need for CD-related surgery

The high prevalence of the *NOD2* p.Leu1007fsX1008 mutation seen in CD patients with an aggressive disease course (e.g., CD patients with a penetrating and/or stricturing disease behavior and/or CD-related surgery) suggests that this mutation is highly associated with a severe CD phenotype. Our sub-analysis emphasizes the strong association of this *NOD2* mutation with severe CD and with ileal involvement (Table 3).

The median age at diagnosis was 25.0 years in *NOD2* wild-type patients (p.Leu1007fsX1008 XX genotype) compared to 22.0 years in heterozygous carriers (p.Leu1007fsX1008 XC genotype) and to 20.0 years in homozygous carriers of the minor C allele (p.Leu1007fsX1008 CC genotype; p = 0.203 for XX vs. XC genotype and p = 0.251 for XC vs. CC genotype, and p = 0.069 for CC vs XX genotype, respectively). Accordingly, the proportion of patients with age at first diagnosis of CD ≤ 16 years (A1 according to the Montreal classification) was lower in the *NOD2* wild-type group than in the p.Leu1007fsX1008/XC or in the p.Leu1007fsX1008/CC group, although these differences did not reach statistical significance (14.3% of patients with A1 in the wild-type group (XX genotype), compared to 16.2% in the XC group and 22.2% in the CC group, p = 0.207 and p = 0.123, respectively).

Furthermore, our sub-analysis showed that homozygous carriers of the p.Leu1007fsX1008 mutation have a more severe CD phenotype with main localization in the terminal ileum (p = 0.020, respectively, Table 3).

**Fig 1:**
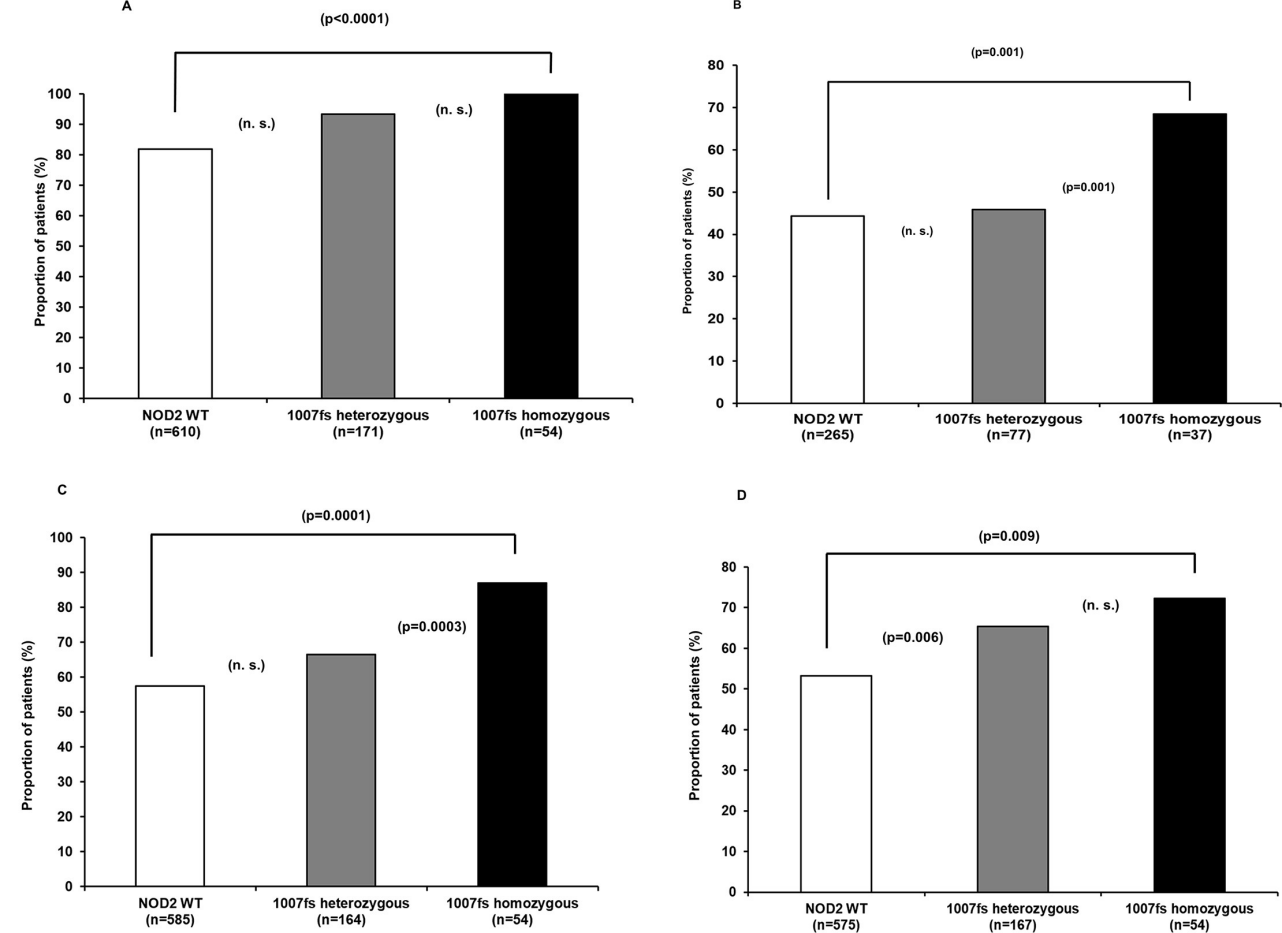
Gene dosage effect of the p.Leu1007fsX1008 mutation on (A) ileal involvement of CD (%), (B) development of fistulas (%), (C) development of stenoses (%), and (D) CD-related surgery (%).

Most strikingly, homozygous carriers of p.Leu1007fsX1008 had significantly more severe disease compared to *NOD2* wild-type carriers (p = 0.003, Table 3). In all homozygous carriers of the CC genotype, the main disease localization was in the terminal ileum compared to 93.6% in heterozygous carriers (XC genotype) and to 82.0% in patients of the *NOD2* wild-type group (Table 3, Fig 1A, p<0.0001 for CC vs. wild-type). 68.5% of the patients with the CC genotype suffered from a penetrating disease (p = 0.001 for CC vs. wild-type), whereas there was no significant difference seen between the wild-type and the XC group (Table 3, Fig 1B, 44.3% vs. 45.8%, p = 0.847). 87% of the patients with homozygosity for p.Leu1007fsX1008 developed intestinal stenoses mainly in the terminal ileum compared to 66.5% of the patients in the XC group and 57.4% in the *NOD2* wild-type group (p = 0.0001 for CC vs. wild-type and p = 0.0.038 for XC vs. wild-type and p = 0.0003 for CC vs. XC genotype, Table 3, Fig 1C). With 72.2%, the highest surgery rate was seen in CC-homozygous patients, compared with 65.3% in heterozygous carriers of the minor C allele (XC genotype) and with 53.2% in the *NOD2* wild-type group (p = 0.118 for CC vs. XC genotype, p = 0.009 CC vs. wild-type and p = 0.006 for XC vs. wild-type, Table 3, Fig 1D).

Overall, 92.6% of the patients with homozygosity for p.Leu1007fsX1008 had either penetrating and/or stricturing disease, while 72.2% needed CD-related surgery (Table 3). In comparison, fewer patients in the *NOD2* wild-type group (72%) and a lower proportion of heterozygous carriers of the minor C allele (XC genotype, 79.2%) had an aggressive disease phenotype (Table 3, p = 0.003 for CC vs. wild-type, p = 0.065 for XC vs. wild-type and p = 0.003 for CC vs. XC genotype).

### Homozygous carriers of p.Leu1007fsX1008 who are active smokers are at 100% risk of developing intestinal stenosis requiring CD-related surgery

An important finding of this study was the strong influence of the *NOD2* genotype and smoking status on the disease course of CD patients. We found a particular strong effect of the p.Leu1007fsX1008 mutation in CD patients with active smoking, resulting in a frequent need for CD related surgery.

**Fig 2:**
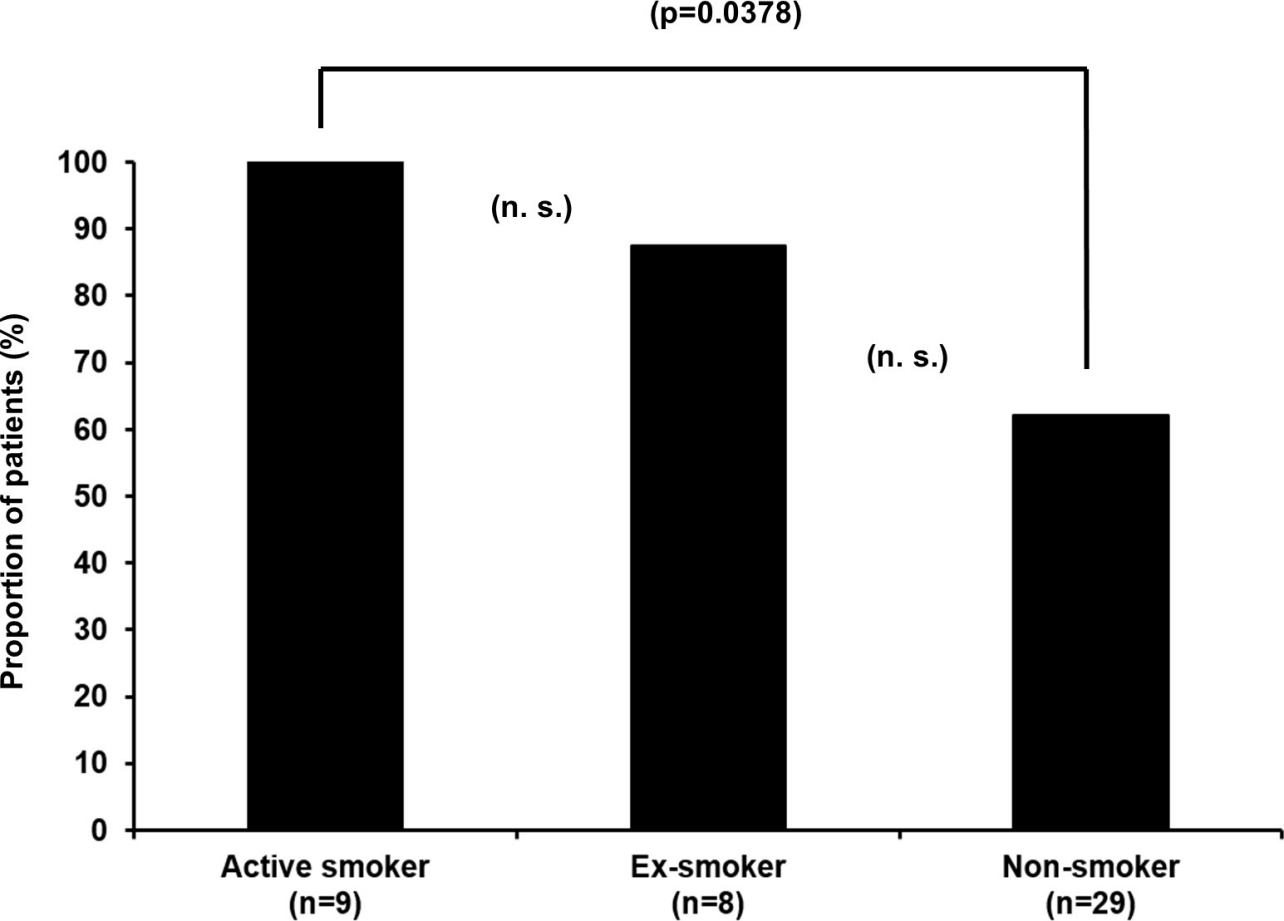
Need for CD-related surgery in homozygous carriers of the p.Leu1007fsX1008 variant based on the smoking history (n = 46).

All patients in the subcohort of CD patients, who were homozygous carriers of p.Leu1007fsX1008 and were active smokers or had a recent smoking history, developed intestinal stenoses, and all these patients needed CD-related surgery (100%, Table 4 and Fig 2). In eight of them (88.9%), an ileocecal resection was performed, and one patient had fistula surgery (Table 4). All patients with a positive smoking history, who stopped smoking during follow-up, also developed intestinal stenosis (100%). The rate of surgery in this group was with 87.5% (n = 7/8) slightly lower compared to active smokers (surgery rate of 100% in active smokers) but higher than compared to non-smokers although this difference was statistically not significant (surgery rate of 62.1% in non-smokers, p = 0.232). In the subcohort of ex-smokers, seven patients with surgical interventions underwent ileocecal resection surgery (Table 4 and Fig 2).

**Table 4:** Risk of development of intestinal stenoses and subsequent need for CD-related surgery with respect to smoking history in homozygous carriers of the minor C allele of p.Leu1007fsX1008.

CD patients with homozygosity for p.Leu1007fsX1008 and CD-related complications based on smoking status (n = 46)				
	Stenoses		Surgery because of CD	
	(n =)	(%)	(n =)	(%)
**Smokers** (n = 9)	9	100.0	9	100.0
**Ex-smokers** (n = 8)	8	100.0	7	87.5
**Non-smokers** (n = 29)	23	79.3	18	62.1

Overall, active smoking in patients with homozygosity for p.Leu1007fsX1008 was associated with the development of intestinal stenoses and the need for CD-related surgery in all cases. However, seven patients (all of them being non-smokers) with homozygosity for p.Leu1007fsX1008 (13.0%) did not develop intestinal stenoses. All of them received immunosuppressive therapy, and they were younger than 40 years at first diagnosis of CD. This suggests that early immunosuppressive therapy may alter the disease course in this patient subcohort.

### Multiple logistic regression analysis confirms homozygosity for the *NOD2* p.Leu1007fsX1008 mutation as major predictor for ileal stenosis and CD-related surgery

To analyze the specific contribution of homozygosity for the p.Leu1007fsX1008 *NOD2* mutation on the risk for intestinal stenosis and CD-related surgery, we performed a multiple logistic regression analysis including predictors for which an effect on the development of CD-related intestinal stenoses and CD-related surgery has been demonstrated in previous studies. The combined effects of five important predictors (age at diagnosis, disease duration, smoking status, ileal involvement and homozygosity for the p.Leu1007fsX1008 *NOD2* mutation (rs2066847)) on the presence of stenoses and on the need for CD-related surgery were analyzed by using multiple logistic regression as detailed in the Methods section. The results from multiple logistic regression for the association of the outcomes "stenoses" or "CD-related surgery" with the five predictors mentioned above are shown in Tables 5 and 6 and S2 and S3 Tables. In these analyses, smoking was represented by one (Tables 5 and 6) or by two contrasts (S2 and S3 Tables).

**Table 5:** Multiple logistic regression analysis including five important predictors (age at diagnosis, disease duration, smoking status, ileal involvement and homozygosity for the p.Leu1007fsX1008 *NOD2* mutation (rs2066847)) on the presence of stenoses.

Variable	p-value	OR (95% CI)
**Age at diagnosis** (per 10 years)	0.447	1.084 [0.881–1.334]
**Disease duration** (per 10 years)	**<0.001**	1.934 [1.456–2.570]
**Smoking status** (any smoking versus non-smoking)	**0.007**	1.968 [1.207–3.208]
**Disease localization** (any ileal involvement vs. none)	**0.027**	2.122 [1.090–4.130]
**Homozygosity for the *NOD2* p.Leu1007fsX1008 mutation** (yes vs. no)	**0.009**	69.533 [2.945–1641.48]

Smoking is represented by a single contrast; the outcome variable is "stenosis" (n = 639). Significant p-values are shown in bold numbers.

**Table 6:** Multiple logistic regression analysis including five important predictors (age at diagnosis, disease duration, smoking status, ileal involvement and homozygosity for the p.Leu1007fsX1008 *NOD2* mutation (rs2066847)) on the need for CD-related surgery.

Variable	p-value	OR (95% CI)
**Age at diagnosis** (per 10 years)	**0.013**	1.309 [1.060–1.617]
**Disease duration** (per 10 years)	**<0.001**	2.793 [2.059–3.789]
**Smoking status** (any smoking versus non-smoking)	0.117	1.487 [0.906–2.440]
**Disease localization** (any ileal involvement vs. none)	0.213	1.571 [0.772–3.196]
**Homozygosity for the *NOD2* p.Leu1007fsX1008 mutation** (yes vs. no)	**0.005**	4.297 [1.557–11.863]

Smoking is represented by a single contrast; the outcome variable is "CD-related surgery" (n = 629). Significant p-values are shown in bold numbers.

Our analyses confirmed the majority of previously known predictors of intestinal stenoses and CD-related surgery in our study cohort (Tables 5 and 6 and S2 and S3 Tables) but showed particular strong associations between homozygosity for the p.Leu1007fsX1008 *NOD2* mutation and stenosis and CD-related surgery, respectively. We demonstrated that the odds ratios for the association of homozygosity for the p.Leu1007fsX1008 *NOD2* mutation with stenoses or with CD-related surgery calculated from the models were larger than in univariable analyses and even larger than for all other previously known predictors of stenosis and CD-related surgery shown in Tables 5 and 6 and S2 and S3 Tables. A very strong association was found between *NOD2* 1007fs homozygosity and intestinal stenoses with an OR > 60 in both models applied (Table 5 and S2 Table). The higher odds ratios in the multivariate analysis (for *NOD2* 1007f homozygosity) are likely due to the fact that *NOD2* 1007fs homozygotes were more often non-smokers than *NOD2* wildtype carriers (Table 4). The negative association between the two risk factors (homozygosity and smoking) would reduce the apparent strength of their association with stenoses or surgery when either predictor is considered separately.

To rule out an interaction between homozygosity for the *NOD2* p.Leu1007fsX1008 mutation and smoking on the presence of stenoses or on the need for CD-related surgery, we performed three-way contingency tables by smoking status, homozygosity for rs2066847 and ileal stenosis (S4A Table) and CD-related surgery (S4B Table). There was no significant interaction for presence of ileal stenoses (p = 1.00) and for the need for CD-related surgery (p = 0.46). Thus, the effects of homozygosity for rs2066847 and smoking on the outcomes (ileal stenoses, CD-related surgery) did not depend on each other and the effect of smoking (increasing the risk of development of CD-related stenoses) was similar in patients with homozygosity for rs2066847 and in other CD patients (S4 Table).

In conclusion, these multivariate analyses strongly support our findings of *NOD2* 1007fs homozygosity as a major predictor of ileal stenosis and CD-related surgery.

## Discussion

The main aim of this study was to evaluate the predictive power of the three major *NOD2* mutations in combination with the smoking status regarding a complicated CD phenotype in a large patient cohort. Our study demonstrates that the *NOD2* mutation p.Leu1007fsX1008, in particular homozygosity for the minor C allele, is an excellent genetic predictor for a complicated disease course in CD patients. Concomitant homozygosity and active smoking carry a 100% risk for developing ileal stenosis and a 100% risk for CD-related surgery. The very high predictive value of p.Leu1007fsX1008 homozygosity is important for guiding therapeutic decisions, such as the early initiation of an immunosuppressive therapy. This is supported by the fact that all homozygous p.Leu1007fsX1008 carriers, who did not develop ileal stenosis, received immunosuppressive therapy. Since 2001, numerous genetic studies on the IBD susceptibility were performed. However, no other genetic marker was identified as having the same risk effect on CD susceptibility and disease severity in adult Caucasian CD patients like *NOD2* [5–23].

To date, our study represents the largest detailed genotype-phenotype analysis of p.Leu1007fsX1008 homozygous CD patients. Our results are in line with a meta-analysis of genome-wide association scans, followed by an extensive validation of significant findings, with a combined total of more than 75,000 cases and controls, which identified a total of 163 IBD loci that met genome-wide significance thresholds [22]. This study confirmed *NOD2* as the main genetic marker for CD but did not provide a detailed phenotype analysis of p.Leu1007fsX1008 homozygous CD patients. Similarly, the IBDchip European Project, a retrospective multicenter cohort study, investigated the influence of CD-related SNPs on the clinical course of CD [18]. Among several SNPs included in the analysis, *NOD2* was the most important genetic factor, being an independent predictive factor for ileal disease location, stenosing and penetrating disease, and the need for surgery and was as such also the strongest factor associated with a complicated disease course [18]. In our study, all 54 1007fs homozygous patients had ileal involvement, with penetrating disease behaviour in more than two thirds of the patients. The surgery rate was 72.2% with ileocecal resection as the main CD-related surgery in almost 85% of patients. More than one fifth of the patients were 16 years or younger at first diagnosis, with the youngest patients being six years old at the time of CD diagnosis. This demonstrates that homozygosity for p.Leu1007fsX1008 is a strong predictor for a severe CD disease course with an early disease onset. Only seven homozygous patients did not develop intestinal stenoses. They all received immunosuppressive therapy. However, their number is too small to draw final conclusions on the effect of immunosuppressive therapy in this patient subcohort but this observation suggests that the early start of an immunosuppressive therapy in these individuals (median of 14 months after first CD diagnosis) may have prevented the development of stricturing disease.

Our results are supported by the largest genotype-phenotype analysis performed in IBD patients which included a total of almost 30 000 IBD patients. In total, 156154 genetic variants were tested for genotype-phenotype associations while only three loci (*NOD2*, MHC, and 3p21) were associated with subphenotypes of IBD and *NOD2* being strongly associated with a severe CD subphenotype [32].

Importantly, in our subcohort of patients with homozygosity for p.Leu1007fsX1008 and a history of active smoking, we demonstrated a uniform, very severe CD phenotype and an association with the development of intestinal stenoses and the need of CD-related surgery in all patients of this subcohort (100%). Similarly, when the subjects had stopped smoking during follow-up, this still was associated with the development of intestinal stenoses in all cases. To our knowledge, this represents the first detailed report on a strong association of *NOD2* p.Leu1007fsX1008 homozygosity in combination with smoking and the subsequent CD disease course. Our findings are strongly supported by the results of a detailed multivariate analysis in which the effect size of 1007fs homozygosity on the development of stenosis and need for CD-related surgery was higher than that of other known predictors of intestinal stenosis and CD-related surgery such as disease duration and smoking status.

Van der Heide et al. suggested an association of p.Arg702Trp (rs2066844) with active smoking in CD and of p.Leu1007fsX1008 and p.Gly908Arg (rs2066845) with non-passive smoking [33]. Further clinical trials showed that smoking is an independent risk factor associated with an unfavorable outcome in CD patients [34–39]. The mechanism by which smoking alters the disease course has not been elucidated yet, but the existing evidence suggests an influence of smoking on the innate and acquired immune system and the intestinal microbiome which is supported by our findings pointing to *NOD2* as a major co-variant by which smoking results in intestinal stenosis [34,36,39–43].

Interestingly, despite adequate knowledge on the association of smoking and an unfavorable CD outcome in the majority of CD patients, more than one third of patients (35.5%) are still active smokers [37]. Furthermore, smoking seems to be associated with higher societal costs and lower quality of life in IBD patients [37]. Thereby, smoking cessation may result in a reduced relapse incidence in CD patients compared to the relapse risk in non-smokers and may result in considerably lower societal costs [37,38].

However, the current study has some intrinsic limitations. The high proportion of patients in our CD cohort with “aggressive” CD (defined as stricturing or penetrating disease behaviour), which often requires CD related-surgery, may be also related to a typical referral center population in our specialized IBD center. Furthermore, the high prevalence of *NOD2* mutations may be explained by the predominantly Caucasian population in our IBD cohort (99.5% Caucasians). In contrast, the association between *NOD2* mutations and CD could not been demonstrated for Asian populations [44,45]. Furthermore, genetic testing focused on the three main *NOD2* mutations, while rare *NOD2* variants were not addressed in this study which would be possible using other techniques such as direct DNA sequencing techniques or genome-wide association scans (GWAS). Therefore, compound heterozygosity of *NOD2* with other risk alleles was not addressed in this current study.

In conclusion, despite growing insights into the genetic background of IBD, *NOD2* is still the most important genetic risk factor for CD. Our results demonstrate that in particular the *NOD2* frameshift mutation p.Leu1007fsX1008 is a strong predictor for a complicated CD course, which requires an early medical intervention in order to prevent CD-related complications. The risk for complicated CD was particularly pronounced in homozygous carriers of the p.Leu1007fsX1008 mutation with a history of active smoking who all developed ileal stenoses and all required CD-related surgery. Given the strong predictive power of active smoking and 1007fs homozygosity for ileal stenosis and CD-related surgery, this *NOD2* variant is a very useful biomarker for clinical and therapeutic decision making on an individual patient level. Our findings are an important step for initiating a more personalized therapy in CD and delivering precision medicine to these patients [19].

## Supporting information

S1 Table Demographic characteristics of the CD study population based on the Montreal classification [25]. For each variable, the number of patients included is given. ^1^ Disease behaviour was defined according to the Montreal classification [25]. A stricturing disease phenotype was defined as the presence of stenoses without penetrating disease. The diagnosis of stenoses was made surgically, endoscopically, or radiologically (using MR enteroclysis). ^2^ Clinical course of CD was furthermore defined as “aggressive” in CD patients with a stricturing and/or penetrating disease behaviour and/or when CD-related surgery became necessary. Accordingly, a “mild” CD phenotype was defined as non-stricturing, non-fistulizing CD without CD-related surgery. ^3^ Immunosuppressive agents included azathioprine, 6-mercaptopurine, methotrexate, infliximab and/or adalimumab^. 4^ Only surgery related to CD-specific problems (e.g. fistulectomy, colectomy, ileostomy) was included.
(DOCX)

S2 Table Multiple logistic regression analysis including five important predictors (age at diagnosis, disease duration, smoking status, ileal involvement and homozygosity for the p.Leu1007fsX1008 *NOD2* mutation (rs2066847)) on the presence of stenoses. Smoking is represented by two contrasts; the outcome variable is "stenosis".
(DOCX)

S3 Table Multiple logistic regression analysis including five important predictors (age at diagnosis, disease duration, smoking status, ileal involvement and homozygosity for the p.Leu1007fsX1008 *NOD2* mutation (rs2066847)) on the need for CD-related surgery. Smoking is represented by two contrasts; the outcome variable is "CD-related surgery".
(DOCX)

S4 Table Three-way contingency tables by smoking status, homozygosity for rs2066847 and (A) ileal stenosis and (B) need for CD-related surgery.
(DOCX)
