# Supplementary material for: Development of a uniform, very aggressive disease phenotype in all homozygous carriers of the NOD2 mutation p.Leu1007fsX1008 with Crohn’s disease and active smoking status resulting in ileal stenosis requiring surgery
Source: PLoS One. 2020 Jul 27;15(7):e0236421. doi: 10.1371/journal.pone.0236421 (PMC7384669; doi:10.1371/journal.pone.0236421)
Supplement: S1 Table — For each variable, the number of patients included is given. 1 Disease behaviour was defined according to the Montreal classification [25]. A stricturing disease phenotype was defined as the presence of stenoses without penetrating disease. The diagnosis of stenoses was made surgically, endoscopically, or radiologically (using MR enteroclysis). 2 Clinical course of CD was furthermore defined as “aggressive” in CD patients with a stricturing and/or penetrating disease behaviour and/or when CD-related surgery became necessary. Accordingly, a “mild” CD phenotype was defined as non-stricturing, non-fistulizing CD without CD-related surgery. 3 Immunosuppressive agents included azathioprine, 6-mercaptopurine, methotrexate, infliximab and/or adalimumab. 4 Only surgery related to CD-specific problems (e.g. fistulectomy, colectomy, ileostomy) was included. (DOCX) [file pone.0236421.s001.docx]

|  | **Crohn’s disease (n = 1076)** |
| --- | --- |
| **Gender** |  |
| Male (%) | 513 (48.0) |
| Female (%) | 563 (52.0) |
| **Age** (years) |  |
| Mean ± SD | 42.3 ± 14.1 |
| Range | (6-87) |
| **Disease duration (years)** |  |
| Mean ± SD | 12.0 ± 9.4 |
| Range | (0-50) |
| **Body mass index** |  |
| Mean ± SD | 22.5 ± 4.3 |
| Range | (13.1-40.8) |
| Age at diagnosis | (n=1000) |
| ≤16 years (A1) | 161 (16.1%) |
| 17-40 years (A2) | 716 (71.6%) |
| > 40 years (A3) | 123 (12.3%) |
| Location | (n=1031) |
| Terminal ileum (L1) | 226 (22.0%) |
| Colon (L2) | 132 (12.8%) |
| Ileocolon (L3) | 659 (64.0%) |
| Upper GI (L4) | 14 (1.4%) |
| Any ileal involvement (L1+L3) | 885 (85.8%) |
| **Behaviour** ^1^ | (n=1014) |
| Non-stricturing, Non-penetrat. (B1) | 278 (27.4%) |
| Stricturing (B2) | 272 (26.8%) |
| Penetrating (B3) | 464 (45.8%) |
| **Clinical disease course** ^2^ | (n=1011) |
| **aggressive** | 747 (73.9) |
| **mild** | 264 (26.1) |
| **Use of immunosuppressive agents** ^3^ | (n=1016) |
|  | 830 (81.7%) |
| **Surgery because of CD** ^4^ | (n=984) |
|  | 561 (57.0%) |
| **Fistulas** | (n=1014) |
|  | 464 (45.8%) |
| **Perianal fistulas** | (n=1014)  137 (13.5%) |
| **Stenosis** | (n=996) |
|  | 605 (60.7%) |
| **Positive family history of IBD** | (n=660) |
|  | 117 (17.7%) |
| **Smoking history** | (n=668) |
| **Active smoker** | 237 (35.5%) |
| **Ex-smoker** | 146 (21.8%) |
| **Non-smoker** | 285 (42.7%) |

**Supplemental table S1.** Demographic characteristics of the CD study population based on the Montreal classification([25](#_ENREF_25)). For each variable, the number of patients included is given.

^1^ Disease behaviour was defined according to the Montreal classification([25](#_ENREF_25)). A stricturing disease phenotype was defined as the presence of stenoses without penetrating disease. The diagnosis of stenoses was made surgically, endoscopically, or radiologically (using MR enteroclysis).

^2^ Clinical course of CD was furthermore defined as “aggressive” in CD patients with a stricturing and/or penetrating disease behaviour and/or when CD-related surgery became necessary. Accordingly, a “mild” CD phenotype was defined as non-stricturing, non-fistulizing CD without CD-related surgery.

^3^ Immunosuppressive agents included azathioprine, 6-mercaptopurine, methotrexate, infliximab and/or adalimumab

^4^ Only surgery related to CD-specific problems (e.g. fistulectomy, colectomy, ileostomy) was included.
