# Supplementary material for: Development of a uniform, very aggressive disease phenotype in all homozygous carriers of the NOD2 mutation p.Leu1007fsX1008 with Crohn’s disease and active smoking status resulting in ileal stenosis requiring surgery
Source: PLoS One. 2020 Jul 27;15(7):e0236421. doi: 10.1371/journal.pone.0236421 (PMC7384669; doi:10.1371/journal.pone.0236421)
Supplement: S2 Table — Smoking is represented by two contrasts; the outcome variable is "stenosis". (DOCX) [file pone.0236421.s002.docx]

| **Variable** | **p-value** | **OR (95% CI)** |
| --- | --- | --- |
| **Age at diagnosis**  (per 10 years) | 0.552 | 1.066 [0.864 - 1.315] |
| **Disease duration**  (per 10 years) | <0.001 | 1.916 [1.442 - 2.544] |
| **Smoking status**  (active smoking versus non-smoking) | 0.068 | 1.646 [0.964 - 2.811] |
| (former smoking versus non-smoking) | 0.004 | 2.619 [1.362 - 5.034] |
| **Disease localization**  (any ileal involvement vs. none) | 0.022 | 2.202 [1.123 - 4.317] |
| **Homozygosity for the *NOD2* p.Leu1007fsX1008 mutation**  (yes vs. no) | 0.009 | 66.211 [2.833 - 1547.56] |

**Supplemental table S2.** Multiple logistic regression analysis including five important predictors (age at diagnosis, disease duration, smoking status, ileal involvement and homozygosity for the p.Leu1007fsX1008 *NOD2* mutation (rs2066847)) on the presence of stenoses. Smoking is represented by two contrasts; the outcome variable is "stenosis".
