# Supplementary material for: Development of a uniform, very aggressive disease phenotype in all homozygous carriers of the NOD2 mutation p.Leu1007fsX1008 with Crohn’s disease and active smoking status resulting in ileal stenosis requiring surgery
Source: PLoS One. 2020 Jul 27;15(7):e0236421. doi: 10.1371/journal.pone.0236421 (PMC7384669; doi:10.1371/journal.pone.0236421)
Supplement: S3 Table — Smoking is represented by two contrasts; the outcome variable is "CD-related surgery". (DOCX) [file pone.0236421.s003.docx]

| **Variable** | **p-value** | **OR (95% CI)** |
| --- | --- | --- |
| **Age at diagnosis**  (per 10 years) | 0.018 | 1.293 [1.045 - 1.601] |
| **Disease duration**  (per 10 years) | <0.001 | 2.770 [2.042 - 3.756] |
| **Smoking status**  (active smoking versus non-smoking) | 0.363 | 1.288 [0.747 - 2.219] |
| (former smoking versus non-smoking) | 0.059 | 1.857 [0.977 - 3.528] |
| **Disease localization**  (any ileal involvement vs. none) | 0.189 | 1.618 [0.789 - 3.314] |
| **Homozygosity for the *NOD2* p.Leu1007fsX1008 mutation**  (yes vs. no) | 0.006 | 4.135 [1.496 - 11.433] |

**Supplemental table S3.** Multiple logistic regression analysis including five important predictors (age at diagnosis, disease duration, smoking status, ileal involvement and homozygosity for the p.Leu1007fsX1008 *NOD2* mutation (rs2066847)) on the need for CD-related surgery. Smoking is represented by two contrasts, the outcome variable is "CD-related surgery".
