# Supplementary material for: Development of a uniform, very aggressive disease phenotype in all homozygous carriers of the NOD2 mutation p.Leu1007fsX1008 with Crohn’s disease and active smoking status resulting in ileal stenosis requiring surgery
Source: PLoS One. 2020 Jul 27;15(7):e0236421. doi: 10.1371/journal.pone.0236421 (PMC7384669; doi:10.1371/journal.pone.0236421)
Supplement: S4 Table — Three-way contingency tables by smoking status, homozygosity for rs2066847 and (A) ileal stenosis and (B) need for CD-related surgery. (DOCX) [file pone.0236421.s004.docx]

**Supplemental table S4.** Three-way contingency tables by smoking status, homozygosity for rs2066847 and **(A)**  ileal stenosis and (B) need for CD-related surgery.

**(A)**

| **Homozygosity for rs2066847** | **Smoking status** | **number of patients** | | **% with**  **ileal stenosis** |
| --- | --- | --- | --- | --- |
|  |  | **no ileal stenosis** | **ileal stenosis** |  |
| no | non-smoker | 70 | 74 | 51.4 |
| no | current smoker | 49 | 92 | 65.2 |
| no | former smoker | 18 | 57 | 76.0 |
| yes | non-smoker | 3 | 22 | 88.0 |
| yes | current smoker | 0 | 9 | 100.0 |
| yes | former smoker | 0 | 8 | 100.0 |

**(B)**

| **Homozygosity for rs2066847** | **Smoking status** | **number of patients** | | **% with**  **CD-related surgery** |
| --- | --- | --- | --- | --- |
|  |  | **no surgery** | **with surgery** |  |
| no | non-smoker | 65 | 76 | 53.9 |
| no | current smoker | 61 | 78 | 56.1 |
| no | former smoker | 21 | 52 | 71.2 |
| yes | non-smoker | 8 | 17 | 68.0 |
| yes | current smoker | 0 | 9 | 100.0 |
| yes | former smoker | 1 | 7 | 87.5 |
